# Levetiracetam alleviates cognitive decline in Alzheimer’s disease animal model by ameliorating the dysfunction of the neuronal network

**DOI:** 10.3389/fnagi.2022.888784

**Published:** 2022-08-25

**Authors:** Xiang-Yu Zheng, Hai-Chen Zhang, Yu-Dan Lv, Feng-Yan Jin, Xiu-Juan Wu, Jie Zhu, Yang Ruan

**Affiliations:** ^1^Department of Neurology and Neuroscience Center, The First Hospital of Jilin University, Changchun, China; ^2^Department of Hematology, Cancer Center, The First Hospital of Jilin University, Changchun, China; ^3^Department of Neurobiology, Care Sciences and Society, Karolinska Institute, Stockholm, Sweden; ^4^Key Laboratory of Pathobiology, Ministry of Education, College of Basic Medical Sciences, Jilin University, Changchun, China

**Keywords:** neuronal dystrophy, neuroinflammation, inflammasome, amyloid β-peptide, dementia

## Abstract

**Background:**

Patients with Alzheimer’s disease (AD) have a significantly higher risk of seizures than other individuals in an age-matched population, suggesting a close association between epilepsy and AD. We aimed to examine the effects of levetiracetam (LEV)—a drug for treating seizures—on learning and memory and the neuropathological features of AD.

**Methods:**

We crossbred APP23 mice with microtubule-associated protein tau (MAPT) transgenic mice to generate APP23/MAPT mice. These mice were treated with different concentrations of LEV in the presence of kainic acid (KA) for 3 months.

**Results:**

Low doses of LEV alleviated the effects of KA on memory defects in APP23/MAPT mice. Mechanistic investigations showed that low concentrations of LEV decreased tau phosphorylation by reducing the activities of cyclin-dependent kinase 5 and glycogen synthase kinase 3α/β, thus rescuing neurons from synaptic dystrophy and apoptosis. Low doses of LEV inhibited the effects of KA (i.e., inducing neuroinflammation and impairing the autophagy of amyloid β-peptide), thus improving cognitive decline. High concentrations of LEV decreased the production and deposition of amyloid β-peptide (Aβ) by reducing the expression of β-site APP-cleaving enzyme 1 and presenilin 1. However, high concentrations of LEV also induced neuronal apoptosis, decreased movement ability in mice, and did not alleviate cognitive decline in AD mice.

**Conclusion:**

Our results support the hypothesis that aberrant network activity contributes to the synaptic and cognitive deficits in APP23/MAPT mice. A low concentration of LEV may help ameliorate abnormalities of AD; however, a high LEV concentration did not induce similar results.

## Introduction

Alzheimer’s disease (AD)—one of the most common causes of dementia—is pathologically characterized by multiple factors, such as the presence of amyloid β-peptide (Aβ) and tau protein in the brain, especially in the hippocampus (Ruan et al., 2019; Zheng et al., 2019). For the production of Aβ, amyloid precursor protein (APP) is sequentially cleaved by β-secretase and γ-secretase during the progression of AD (O’Brien and Wong, 2011). Tau is also hyperphosphorylated, leading to the destabilization of the microtubules in neurons. With the progression of AD, Aβ and tau form aggregates that are deposited in senile plaques (SPs) and neurofibrillary tangles (Hardy, 2006), potentially contributing to the pathogenesis of AD (Bennett et al., 2004).

There are no effective therapies for preventing or reversing AD and it is predicted that by 2050 the number of AD patients will increase to 100 million worldwide (Wimo and Prince, 2010). Therefore, identifying suitable therapies is an urgent matter. A series of clinical trials have tested the possibilities of anti-Aβ therapies (Golde et al., 2011; Huang and Mucke, 2012). However, the efficacy and safety of these treatments are uncertain because of the serious side effects of blocking Aβ production or enhancing its clearance (Golde et al., 2010). Moreover, the perturbation of neuronal network activity by Aβ, phosphorylation of tau, and other factors may be major and early contributors to AD pathogenesis (Palop and Mucke, 2009). Thus, identifying the drugs for maintaining or restoring the activities of neuronal networks may provide a better therapeutic strategy to treat AD.

In this regard, the relationship between epilepsy and AD has already been reported in the past decades. For instance, clinical trials have demonstrated a considerably higher incidence of seizures in AD patients than in matched control subjects (Hesdorffer et al., 1996; Amatniek et al., 2006). Similar results have been observed in AD animals, such as hAPP and APP/PS1 transgenic (Tg) mice (Palop et al., 2007; Minkeviciene et al., 2009). Moreover, ectopic expression of the intracellular domain of APP can potentially contribute to seizure inducing in mice (Vogt et al., 2011). Excessive neuronal excitability may also be involved in the deposition of Aβ in AD animals (Bero et al., 2011). In line with these reports, our previous studies have revealed the key roles of kainic acid (KA)—an analog of the excitotoxin glutamate—in the deposition of Aβ and tau hyperphosphorylation in APP23 and microtubule-associated protein tau (MAPT) Tg mice, which serve as animal models of AD (Ruan et al., 2019; Zheng et al., 2019). Hence, aberrant excitatory activity in neurons has been hypothesized to be the primary mechanism of AD.

Antiepileptic drugs have been shown to be effective in improving cognition in patients with mild cognitive impairment (Bakker et al., 2012). For example, valproic acid is effective in inhibiting the production of Aβ and phosphorylated tau (p-tau), thereby improving the learning and memory abilities of AD animals (Qing et al., 2008; Hu et al., 2011). The therapeutic effects of topiramate (TPM) and levetiracetam (LEV) on AD have also been demonstrated (Eyal et al., 2004). These investigations ascribe the therapeutic effects of antiepileptic drugs to their inhibitory action of histone deacetylase. Furthermore, TPM and LEV provide additional benefits such as neuroprotection and anti-inflammation, which are involved in the pathogenesis of AD (Belcastro et al., 2011; Thöne et al., 2012). However, it remains uncertain whether these antiepileptic drugs can reverse neuropathology and cognitive deficits *via* maintaining or restoring the activities of neuronal networks in AD transgenic mice.

In view of these findings, we used LEV to examine the effects of antiepileptic drugs on AD in KA-stimulated APP23/MAPT mice. We found that chronic treatment with low doses of LEV reversed behavioral abnormalities and cognitive impairments by inhibiting tau phosphorylation, which protects neurons from synaptic dystrophy and apoptosis. Moreover, LEV inhibited neuroinflammation and contributed to Aβ degradation by restoring autophagy, resulting in memory improvement. However, even though high concentrations of LEV blocked the production and deposition of Aβ, they also induced apoptosis in neurons and, thus, did not alleviate the cognitive decline in AD.

## Materials and methods

### Mice and treatment protocol

APP23 [B6-Tg (Thy1APP) 23SdZ] and MAPT [B6; C3-Tg (Prnp-MAPT*P301S) PS19Vle/J] mice were obtained from the Jackson Laboratory (Bar Harbor, ME, United States; Stock #030504 and #008169). APP23 and MAPT mouse lines—which are Tg for human APP and carry the Swedish and P301S mutations, respectively—were generated on the C57BL/6 genetic background using the following methods described elsewhere (Ruan et al., 2019; Zheng et al., 2019). The APP23 mice were crossbred with the MAPT Tg mice to generate APP23/MAPT mice. All of the animal experimental procedures were approved by the Institutional Animal Care and Use Committee of The First Hospital of Jilin University and were in compliance with the Guidelines for the Care and Use of Laboratory Animals of the U.S. National Institutes of Health. The mice were housed five per cage in a room maintained at 22 ± 2°C with an alternating 12-h light-dark cycle. Food and water were available *ad libitum*.

Based on a previous study, six male mice per group at the age 5-month-old were simultaneously treated with an intraperitoneal injection of 10 mg/kg/d KA (Sigma-Aldrich Corp., St. Louis, MO, United States) in the absence or presence of various doses of LEV (50, 100, 150, 200 mg/kg/d) (Aladdin, Shanghai, China) emulsified in 0.9% phosphate-buffered saline [PBS (–)]. The male mice in the control group (*n* = 6) were injected with PBS (–). After 3 months of treatment, behavioral tests were performed in all mice when the treatment is terminated. Following this, the mice were sacrificed and their brain tissue was harvested for further analysis.

### Morris water maze test

To assess any cognitive changes, the Morris water maze (MWM) test was conducted in a circular water tank (diameter, 140 cm; height, 40 cm) filled with water to a depth of 20 cm, maintained at 21 ± 1^°^C. The tank was divided into four equal quadrants. A submerged square platform was placed in the third quadrant of the tank with its top surface 1 cm below the water surface. The mice were placed in the pool at four possible start locations facing the wall of the pool, and a camera was simultaneously activated. Each mouse was allowed up to 60 s to locate the platform. The trial was terminated when the mouse found the platform within 60 s. If a mouse failed to find the platform within 60 s, it was guided by a researcher to locate the platform and allowed to stay for 2–3 s. Each mouse was trained three times per day for 2 days to allow them to adapt to the pool environment (visible platform training) and then underwent the test three times per day for 4 days to test their ability to find the hidden platform. The latency (time taken to locate the platform in the water), distance, and swim speed were recorded using an automated video-tracking software package (EthoVision 2.3.19; Noldus, Wageningen, the Netherlands). On the 7th day, the platform was removed and the number of crossings of the mice from the platform’s original location were tracked and videotaped.

### Open field test

The mice were transferred to the testing room for 1 h before testing to allow them to acclimate to the environment. The mice were tested in a clear plastic cage and the same automated video-tracking software package (EthoVision 2.3.19; Noldus) was used to track their total movements. The movements in the center and periphery of the open field were recorded for further data analysis.

### Tissue procession

After harvesting the brains of mice, the brains were divided into two halves. Half brain was immobilized in 4% paraformaldehyde for 24 h, which was then submerged in 30% sucrose solution before sectioning. The other half of brain was used to extract proteins by a protein extraction kit (Thermo Fisher Scientific, Shanghai, China) following the manufacturer’s protocol.

### Immunochemistry

Mouse brain tissues were frozen and sectioned consecutively into 10-μm sections, with three sections for each embedded sample. The sections were incubated overnight at 4^°^C with Aβ antibody (1:500; Cell Signaling Technology, Danvers, MA, United States). After incubation with horseradish peroxidase-conjugated secondary antibody (Cell Signaling Technology) for 2 h at room temperature, the sections were washed and developed with 0.05% diaminobenzidine plus 0.015% hydrogen peroxide in PBS. The diaminobenzidine-stained sections were air-dried, counterstained with Mayer’s hematoxylin, dehydrated, cleared, and coverslipped. Finally, the sections were visualized with diaminobenzidine for light microscopy examination (Olympus BX50; Center Valley, PA, United States).

### Double immunofluorescence staining

The frozen sections (10-μm thick) of the mice brains were blocked with 5% normal goat serum in tris-buffered saline (TBS) for 30 min, followed by incubation with primary antibodies overnight at 4^°^C in TBS containing 5% goat serum and 0.1% Triton. Primary antibodies used included mouse monoclonal Aβ (1:250), rabbit monoclonal NF-κB p65 (1:50; Cell Signaling Technology), and rabbit monoclonal NLRP3 (1:100; Cell Signaling Technology). Importantly, the antibody used for NF-κB in the subsequent experiments was a probing p65 subunit. After washing with TBS, the sections were incubated with anti-mouse IgG (H + L chains) F(ab’)_2_ fragment (Alexa Fluor 488 Conjugate) (1:1,000; Cell Signaling Technology) and anti-rabbit IgG (H + L) F(ab’)_2_ fragment (Alexa Fluor 594 Conjugate) (1:1,000; Cell Signaling Technology) in TBS containing 5% goat serum and 0.1% Triton for 1 h at room temperature. The double immunostaining results were analyzed using a laser scanning confocal microscope (A1; Nikon, Shanghai, China).

### Western blotting

Total protein was extracted using a protein extraction kit (Thermo Fisher Scientific, Shanghai, China) following the manufacturer’s protocol. Protein extracts were dissolved in 10–15% sodium dodecyl-sulfate polyacrylamide gel electrophoresis and then transferred to a polyvinylidene fluoride membrane at 100 V for 1 h. The membrane containing protein extracts was blocked with 5% non-fat skim milk diluted with TBS containing 0.1% Tween 20 (TBST) for 1 h at room temperature and then incubated overnight with primary antibody (diluted with 2% bovine serum albumin in TBST) at 4^°^C. The following primary antibodies were used: anti-β-actin (1:5,000), anti-p-NF-κB (1:2,000), anti-NLRP3 (1:3,000), anti-IL-1β (1:1,000), anti-ADAM10 (1:1,000), anti-β site APP cleavage enzyme 1 (BACE1) (1:2,000), anti-presenilin 1 (PS1) (1:1,000), anti-p-APP (1:1,000), anti-APP (1:2000), anti-Aβ (1:1,000), anti-Beclin1 (1:2,000), anti-LC3 (1:2,000), anti-LRP1 (1:5,000), anti-RAGE (1:3,000), anti-p-glycogen synthase kinase 3α/β (GSK3α/β) (1:4,000), anti-GSK3α/β (1:4000), anti-p-cyclin-dependent kinase 5 (CDK5) (1:4,000), anti-CDK5 (1:4,000), anti-p-tau (1:2,000), anti-GFAP (1:3,000), anti-Iba1 (1:1,000), anti-SYP (1:500), anti-PSD95 (1:2,000), anti-NeuN (1:2,000), anti-Bax (1:2,000), and anti-Bcl-2 (1:2,000) (Cell Signaling Technology); anti-sAPPα (1:500) and anti-sAPPβ (1:500) (Immuno-Biological Laboratories, Naka Aza-Higashida, Fujioka-Shi, Japan); and anti-APOE (1:2,000), anti-IDE (1:1,000), and anti-NEP (1:500) (Santa Cruz Biotechnology, Shanghai China). On the second day, the proteins were visualized using an enhanced chemiluminescence detection system (Thermo Fisher Scientific) after incubating with corresponding secondary antibodies (1:10,000, Cell Signaling Technology) and visualized using Bio-Rad ChemiDoc XRS devices (Bio-Rad Laboratories, Shanghai, China). For the quantitative analysis of the band intensity, we measured the band intensity ratio for normalization using the ImageJ software. For each blot, we multiplied the background-subtracted density of the target protein in each lane with the ratio of the density of the loading control (such as a housekeeping protein) from a control sample in all of the study blots to the other lanes in the gel. This provided a normalized density with respect to the loading control (NDL). We calculated the fold difference for each replicate by dividing the NDL from each lane by the NDL from the control sample.

### Enzyme activity assay

For sandwich enzyme-linked immunosorbent assay (ELISA), a 96-well plate (MaxiSorp, Nunc, Denmark) was coated with 100 μl of Aβ42-specific antibody (5.0 μg IgG/mL each) overnight at 4^°^C in 100 mM carbonate buffer (pH 9.6) containing 0.05% sodium azide. After blocking with 1% Block Ace in PBS overnight at 4^°^C, the standards (human synthetic Aβ peptides 1–42) and samples were loaded and incubated overnight at 4^°^C. Horseradish peroxidase-conjugated detection antibody was incubated for 4 h at room temperature and visualized using a TMB substrate.

### Survival rate

In total, 1 × 10^4^ N2a mouse neuroblastoma cell line cells were cultured in 96 well plates in a humidified incubator in the presence of 5% CO_2_ at 37^°^C. The Dulbecco’s Modified Eagle Medium (Gibco, Grand Island, NY, United States) with 10% fetal bovine serum (Gibco) was replaced when its color turned yellow. After treatment with KA (10 μM) in the absence or presence of indicated concentrations of LEV (50, 100, 150, or 200 μM) for 24 h, the survival rate of cells was determined using an MTT assay kit (Abcam, Shanghai, China). In brief, 50 μL of serum-free medium and 50 μL of MTT reagent were added into each well after discarding the treatment medium. The plate was incubated at 37^°^C for 3 h. Then, the MTT reagent-supplement medium was removed and 150 μL of MTT solvent was added into each well. After wrapping the plate in foil and shaking it on an orbital shaker for 15 min, the absorbance at 590 nm was measured. The survival rate was calculated according to the following equation.


$$\%\,S\,u\,r\,v\,i\,v\,a\,l\,r\,a\,t\,e=\frac{100\times \left(O\,D_{c\,o\,n\,t\,r\,o\,l}-O\,D_{s\,a\,m\,p\,l\,e}\right)}{O\,D_{c\,o\,n\,t\,r\,o\,l}}$$

### Statistical analysis

The data are expressed as the mean ± standard deviation and analyzed using the SPSS 10.0 statistical software (SPSS Inc., Chicago, IL, United States). One-way and two-way analysis of variance (ANOVA) tests were used to determine the significance of the differences among the groups (*P* < 0.05, *P* < 0.01, and *P* < 0.001).

## Results

### Low levetiracetam concentration improves kainic acid -impaired learning and memory ability in APP23/microtubule-associated protein tau mice; however, a high concentration of levetiracetam did not induce similar results.

**FIGURE 1:**
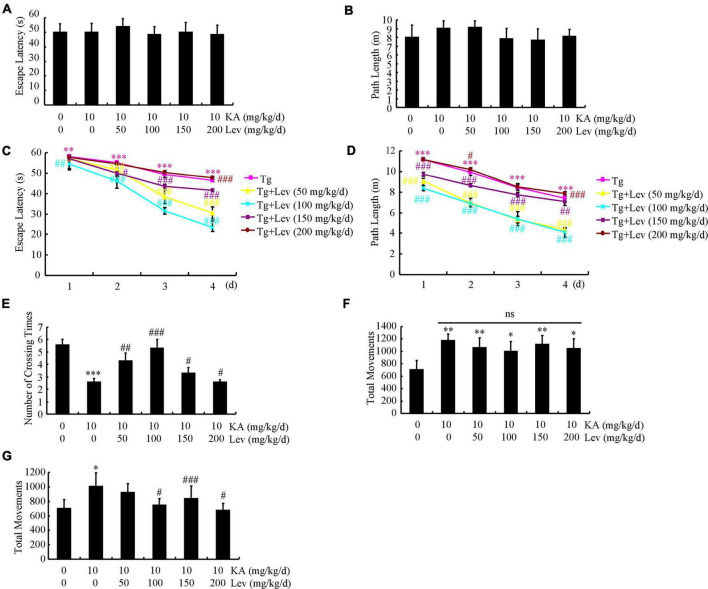
LEV mitigates KA-induced behavioral abnormalities in APP23/MAPT mice. **(A,B)** During the first 2 days of visible platform tests, the KA- and LEV-treated and control APP23/MAPT mice exhibit similar latencies in escape time (*P* > 0.05, ANOVA). **(C,D)** In the hidden platform tests, KA-treated APP23/MAPT mice show a longer latency period and travel distance to escape on days 3 and 4; this is ameliorated by treatment with low concentrations of LEV on day 4 (***P < 0.001 vs. the control group; ^#^*P* < 0.05; ^##^*P* < 0.01; ^###^*P* < 0.001 vs. the KA-treated group by ANOVA). **(E)** In the probe trial on day 7, the KA-treated APP23/MAPT mice travel into the third quadrant (where the hidden platform was previously placed) in significantly shorter times than controls, after treatment with LEV (***P < 0.001 vs. the control group; ^#^*P* < 0.05; ^##^*P* < 0.01; ^###^*P* < 0.001 vs. the KA-treated group by ANOVA). **(F,G)** Total movements of mice in different groups tested in the open field experiments (*P < 0.05; ^**^P < 0.01; ^***^P < 0.001 vs. the control group; ^#^*P* < 0.05; ^###^*P* < 0.001 vs. the KA-treated group by ANOVA) KA, kainic acid; LEV, levetiracetam.

Given the potential therapeutic effects of LEV on AD (Eyal et al., 2004), an MWM test was carried out to analyze the memory loss in the mice. On the first 2 days of assays, the mice did not show any defect demonstrating similar escape latency and path length to the visible platform (Figures 1A,B). In comparison, KA treatment increased the mean escape latency of the mice, which was ameliorated by adding LEV in a low doses [LEV (L)] (Figure 1C). Consistently, the path length was also increased in the KA-treated group, which was decreased by the addition of LEV (L) (Figure 1D). On the 7th day, the probe trial was conducted by removing the platform. As expected, the crossing times were shorter in KA-treated mice, which were partially recovered by LEV (L) treatment (Figure 1E). Notably, these results were not reproduced in groups treated with a high concentration of LEV [LEV (H)] (Figures 1C–E). Taken together, these results demonstrated that a low concentration of LEV ameliorated KA-induced memory deficits; however, a high concentration of LEV did not induce similar results.

### Chronic treatment with a high doses of levetiracetam partially reverses hyperactivity of APP23/microtubule-associated protein tau mice

Since APP Tg mice are hyperactive, we proceeded to determine the movements of APP23/MAPT mice in the open field test. The mice were then divided into groups in such manner that baseline activity levels did not differ between genotype-matched groups that received KA treatment (10 mg/kg/d), even though KA obviously elevated the movement frequency of mice (Figure 1F). With the chronic administration of different LEV concentrations for 3 months, we continued to test the movements of APP23/MAPT mice in different groups. LEV treatment partially reversed the abnormal increase in the movements of APP23/MAPT mice in the periphery of the open field, which accounted for most of their hyperactivity in this paradigm (Figure 1G).

### A high levetiracetam concentration mitigates kainic acid -induced production and aggregation of aβs by inhibiting the amyloidal procession of amyloid precursor protein; a low concentration does not induce the same results

**FIGURE 2:**
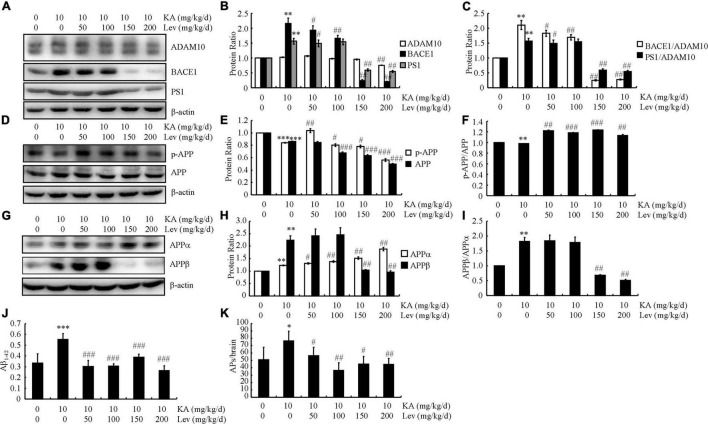
High concentrations of LEV suppress the production and deposition of Aβ in KA-stimulated APP23/MAPT mice. **(A–C)** Protein levels of ADAM10, BACE1, and PS1 in the brains of KA (10 mg/kg/d)- or LEV (50, 100, 150 or 200 mg/kg) + KA-treated APP23/MAPT mice. **(D–F)** Protein levels of p-APP and APP in the brains of KA- or LEV (50, 100, 150, or 200 mg/kg) + KA-treated APP23/MAPT mice. **(G–I)** Protein levels of sAPPα and sAPPβ in the brains of KA- or LEV (50, 100, 150, or 200 mg/kg) + KA-treated APP23/MAPT mice. The optical densities of bands in the western blots were evaluated using the ImageJ software. **(J)** The production of Aβ_1–42_, as determined using enzyme-linked immunosorbent assay. **(K)** The number of SPs. As determined by immunohistochemical staining (*P < 0.05, **P < 0.01, and ***P < 0.001 vs. controls; ^#^*P* < 0.05; ^##^*P* < 0.01, and ^###^*P* < 0.001 vs. the KA group; one-way ANOVA). KA, kainic acid; LEV, levetiracetam; SPs, senile plaques; Aβ, amyloid β-peptide.

LEV has been shown to modulate the behaviors of APP23/MAPT mice. Therefore, we evaluated its effects on the production and deposition of Aβ in the brains of APP23/MAPT mice. To this end, we prioritized quantifying the expression levels of α-, β- and γ-secretases in these mice. The protein levels of BACE1 and PS1 were increased in the KA-treated groups (Figures 2A,B). However, after the addition of LEV (H), the expression levels of BACE1 and PS1 were decreased (Figures 2A,B). On calculating the ratio between secretases, we found that the ratios of BACE1/ADAM10 and PS1/ADAM10 were decreased after the addition of LEV (H) in KA-treated APP23/MAPT mice (Figure 2C).

Although KA treatment did not significantly affect the phosphorylated and total protein levels of APP, treatment with LEV (H) slightly suppressed these levels (Figures 2D,E). As both the phosphorylated and total protein levels of APP are downregulated simultaneously, we did not observe a noticeable change in the ratio between phosphorylated and total levels of APP (Figure 2F).

Given the ability of LEV (H) to suppress the expression of BACE1 and PS1, we continued to evaluate the production of sAPPα and sAPPβ. Consistent with the previous observations, the level of sAPPα was slightly upregulated, indicating the processing of APP products mediated by ADAM10 (Figures 2G,H). Moreover, the products of the BACE1-mediated cleavage of APP—namely, sAPPβ—were markedly suppressed in KA-stimulated mice treated with LEV (H) (Figures 2G,H). As a result, the ratio between sAPPβ and sAPPα was lower in mice treated with high concentration thresholds of LEV (Figure 2I).

The aforementioned results suggest that LEV may be involved in modulating Aβ production and deposition. ELISA and immunohistochemistry assays were performed to verify this hypothesis. In the KA-treated groups, the production of Aβ was significantly increased (Figure 2J). However, after LEV treatment, Aβ production decreased significantly (Figure 2J). Consistent with these findings, the aggregation of Aβ in KA-treated mice was significantly decreased after LEV treatment (Figure 2K). Based on these findings, it is worth noting that LEV inhibited Aβ deposition in the KA-treated mice.

### Levetiracetam treatment promotes the clearance of Aβ in kainic acid-treated APP23/microtubule-associated protein tau mice

**FIGURE 3:**
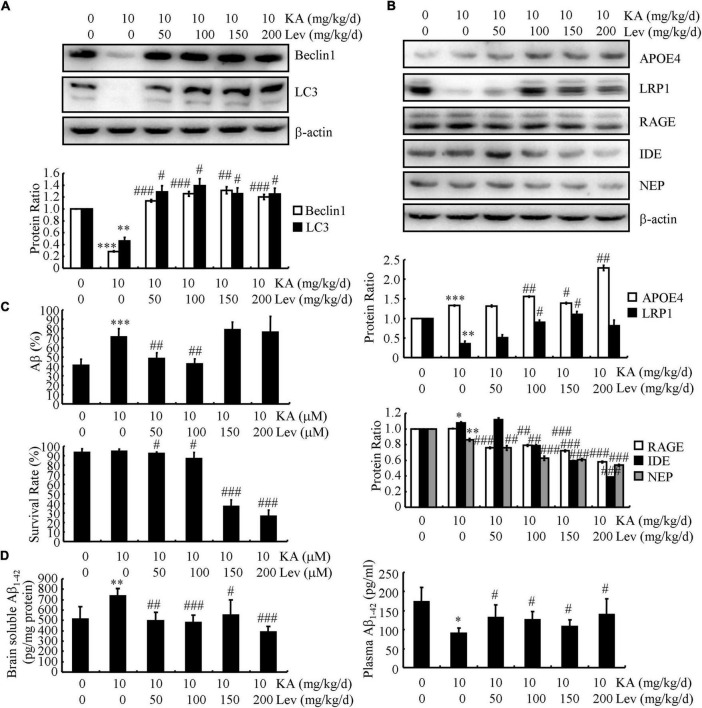
LEV treatment promotes the clearance of Aβ in KA-stimulated APP23/MAPT mice. **(A,B)** Protein levels of Beclin1, LC3, APOE, LRP1, RAGE, IDE, and NEP in the brains of KA (10 mg/kg/d)- or LEV (50, 100, 150, or 200 mg/kg/d) + KA-treated APP23/MAPT mice. **(C)** Mouse neuroblastoma cell line N2a cells are treated with hAβ (10 ng/mL) for 24 h; after incubation with KA and LEV (50, 100, 150, or 200 M), the remaining hAβ-treated cells were assessed using enzyme-linked immunosorbent assay and the survival rate was calculated using the MTT assay. **(D)** hAβ (3 μg) was intracerebroventricularly injected into the mice; after 24 h, the levels of hAβ in the brain and plasma of APP23/MAPT mice were determined using enzyme-linked immunosorbent assay (*P < 0.05; ^**^P < 0.01; ^***^P < 0.001 vs. the control group; ^#^*P* < 0.05; ^##^*P* < 0.01; ^###^*P* < 0.001 vs. the KA-treated group, Student’s *t*-test). KA, kainic acid; LEV, levetiracetam; Aβ, amyloid β-peptide; h, human.

In addition to Aβ production and deposition, Aβ clearance also plays a key role in Aβ homeostasis in the brain. Therefore, we further evaluated the expression of key proteins involved in Aβ degradation and clearance in different groups of mice. On measuring autophagy-associated proteins in the brains of APP23/MAPT Tg mice, we found that KA significantly reduced the levels of Beclin1 and LC3 (western blots, Figure 3A). We further examined the levels of Beclin1 and LC3 in LEV-treated mice. As expected, LEV treatment increased the levels of Beclin1 and LC3 in KA-treated mice (Figure 3A). We also evaluated the expression of key proteins involved in Aβ clearance in these groups. The expression levels of APOE, RAGE, IDE, and NEP did not change significantly in these groups (Figure 3B). However, the expression of LRP1 in the KA-treated mice was significantly lower than that in the control mice (Figure 3B). Addition of LEV attenuated the effects of KA on reducing the expression of LRP1 in the brains of APP23/MAPT mice (Figure 3B), suggesting that LEV may decrease Aβ content partly by promoting Aβ clearance in the brains of KA-treated sAPP23/MAPT mice (Figure 3B).

Moreover, we incubated the N2a cells with human (h) Aβ (10 ng/mL) for 24 h. After incubation with KA, the levels of the remaining hAβ were elevated and then decreased after the addition of LEV (L) (Figure 3C). However, LEV (H) did not show similar effects against KA treatment (Figure 3C); LEV (H)-treated N2a cells did not survive (Figure 3C). These observations suggest that LEV alleviated the effects of KA on impairing the degradation of Aβ *via* autophagy. Furthermore, the mice were administered intracerebroventricular injections of hAβ (3 μg). After 24 h, the levels of hAβ were determined in the brains and plasma of APP23/MAPT mice. The results demonstrated that LEV treatment decreased the levels of Aβ in the brains of KA-treated mice (Figure 3D), whereas LEV enhanced the plasma levels of Aβ in KA-treated mice (Figure 3D). These results suggest that LEV increased the efflux of Aβ from the brains to the plasma in APP23/MAPT mice.

### Levetiracetam dephosphorylates tau *via* the GSK3α/β and CDK5 pathways in kainic acid-treated APP23/microtubule-associated protein tau mice

**FIGURE 4:**
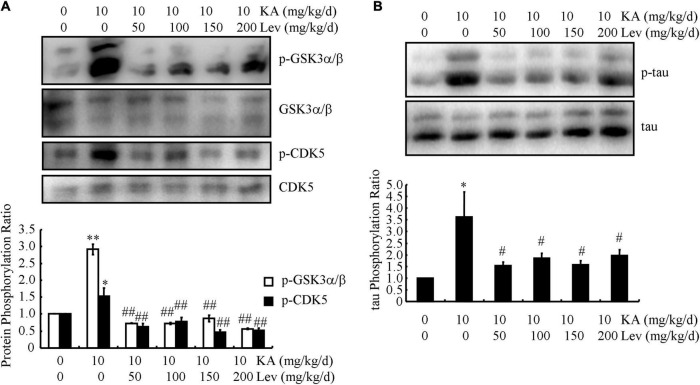
LEV suppresses KA-induced tau phosphorylation in the brains of APP23/MAPT mice. **(A)** Phosphorylated and total protein levels of GSK3α/β and CDK5 in the brains of KA (10 mg/kg/d)- or LEV (50, 100, 150, or 200 mg/kg/d) + KA-treated APP23/MAPT mice, as determined by western blots. **(B)** Phosphorylated and total protein levels of tau in the brains of KA- or LEV (50, 100, 150, or 200 mg/kg) + KA-treated APP23/MAPT mice, as determined by western blots. The optical densities of bands in the western blots were evaluated using the ImageJ software (*P < 0.05; ^**^P < 0.01 vs. the control group; ^#^*P* < 0.05; ^##^*P* < 0.01 vs. the KA-only group, one-way ANOVA). KA, kainic acid; LEV, levetiracetam; Aβ, amyloid β-peptide.

Tau phosphorylation is another remarkable pathological feature of AD. We, therefore, examined the levels of p-tau in different groups of APP/MAPT mice. As shown in Figure 4A, the levels of p-CDK5 and p-GSK3α/β, which are positively associated with enhanced phosphorylation of tau, were markedly increased in the KA-treated mice compared to those in the control mice (Figure 4A). With the addition of LEV, the phosphorylation of CDK5 and GSK3α/β was attenuated in KA-treated mice (Figure 4A), indicating that LEV inhibited the phosphorylation of tau by deactivating CDK5 and GSK3α/β. Based on these observations, the phosphorylation of tau was further evaluated using western blots, and our results revealed that LEV treatment suppressed the phosphorylation of tau in KA-treated APP23/MAPT mice (Figure 4B).

### NF-κB and NLRP3 are colocalized with senile plaques

**FIGURE 5:**
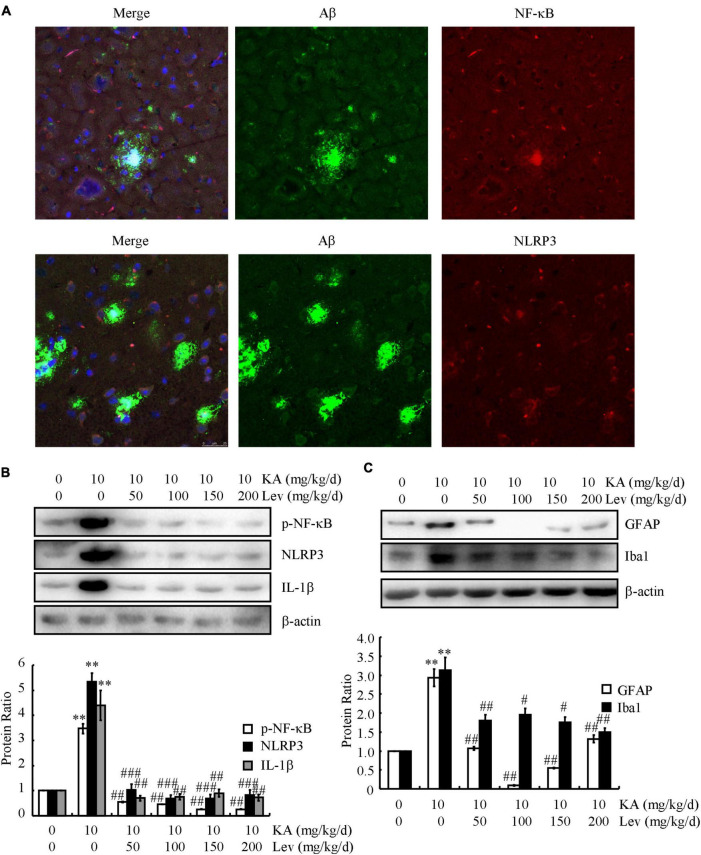
LEV treatment mitigates the effects of KA on activating inflammasomes in the brains of APP23/MAPT mice. **(A)** Images from a single z-plane of the brains of 9-month-old APP23/MAPT mice using monoclonal antibodies against Aβ (green), NF-κB/NLRP3 (red), and the nucleus (blue DAPI stain). **(B,C)** The phosphorylation level of NF-κB and the total protein levels of NLRP3, IL-1β, GFAP, and Iba1 in the brains of KA- or LEV (50, 100, 150, or 200 mg/kg) + KA-treated APP23/MAPT mice, as determined by western blots. The optical densities of bands in the western blots were evaluated using the ImageJ software (^**^P < 0.01 vs. the control group; ^#^*P* < 0.05; ^##^*P* < 0.01; ^###^*P* < 0.001 vs. the KA-only group). KA, kainic acid; LEV, levetiracetam; Aβ, amyloid β-peptide.

Given the potential contribution of inflammasomes to AD, we characterized the relationship between NF-κB/NLRP3 and SPs. Confocal microscopy was used to study the localization of NF-κB/NLRP3 and Aβ in 6-month-old APP23/MAPT mice (Figure 5A). Both NF-κB and NLRP3 were deposited with SPs (Figure 5A), indicating the colocalization of NF-κB and NLRP3 with SPs.

### Levetiracetam treatment mitigates the effects of kainic acid on activating inflammasomes in the brains of APP23/microtubule-associated protein tau mice

Since KA can induce the production and aggregation of Aβ and cause memory deficits by activating inflammasomes in NLRP3- and NF-κB-stimulating pathways, we examined the effects of LEV on inflammasomes. Using western blots, we observed a significant decrease in the phosphorylation of NF-κB and the expression of NLRP3 and IL-1β in the brains of LEV-treated APP23/MAPT mice (Figure 5B). Furthermore, western blots showed that the optical densities of GFAP (astrocyte biomarker) and Iba1 (microglial biomarker) were clearly decreased in the LEV-treated groups compared to those in the KA-treated group (Figure 5C). These results suggest that LEV inhibited inflammatory stress *via* the deactivation of inflammasomes.

### Low concentrations of levetiracetam protect neurons from neuronal dystrophy and neuronal loss, whereas high concentrations induce apoptosis in neurons

**FIGURE 6:**
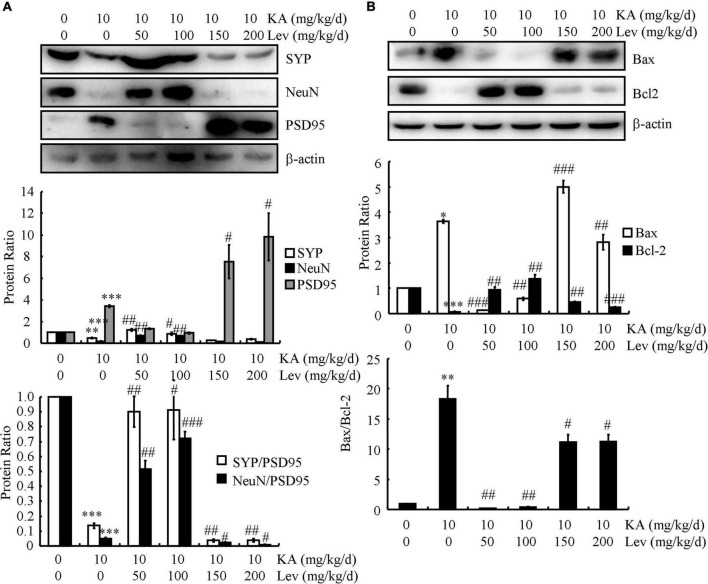
Low doses of LEV protect neurons from neuronal loss, whereas high doses of LEV induce the apoptosis of neurons. **(A,B)** The protein levels of SYP, NeuN, PSD95, Bax, and Bcl2 were determined in the brains of KA (10 mg/kg)- or LEV (50, 100, 150, or 200 mg/kg)+KA-treated APP23/MAPT mice, as determined by western blots. The optical densities of bands in the western blots were evaluated using ImageJ software (*P < 0.05; **P < 0.01; and ***P < 0.001 vs. the control group; ^#^*P* < 0.05; ^##^*P* < 0.01; and ^###^*P* < 0.001 vs. the KA-only group). KA, kainic acid; LEV, levetiracetam.

The accumulation of Aβ and hyperphosphorylated tau results in dystrophy and neuronal loss, which prompted us to examine the effects of LEV on neurons. For this purpose, the expression of the presynaptic marker (SYP) and postsynaptic marker (PSD95) was determined using western blots. The results demonstrated that the expression of SYP was markedly downregulated, whereas PSD95 was dramatically upregulated in the KA-treated mice compared to those in the control subjects (Figure 6A), which indicated the impairment of synaptic plasticity by KA in the brains of APP23/MAPT mice. Consistently, the protein level of a postmitotic neuronal marker (NeuN) was significantly lower in the KA-treated mice than in the control mice (Figure 6A). On the basis of these results, KA was suggested to promote synaptic dystrophy and neuronal loss in the brains of APP23/MAPT mice. Moreover, the addition of LEV (L) attenuated the effects of KA on neuronal dystrophy and neuronal loss (Figure 6A). However, LEV (H) led to neuronal apoptosis by upregulating the expression of Bax and downregulating the expression of Bcl2 in the brains of KA-stimulated APP23/MAPT mice (Figure 6B). This also explains why LEV (H) could not alleviate the cognitive decline in APP23/MAPT mice.

## Discussion

Excessive excitotoxicity has been reported to contribute to the neurodegenerative processes. Glutamate and its associated excitatory amino acids can impair neurons by inducing apoptosis *in vitro* and *in vivo* (Ding et al., 2015). As an analog of glutamate-associated amino acids, KA has recently been reported to be associated with AD, especially with the production and deposition of Aβ (Ruan et al., 2019) and hyperphosphorylation of tau (Zheng et al., 2019). In this study, we extended our previous research to test the effects of an antiepileptic drug, LEV, on memory impairment and neuropathology in APP23/MAPT mice. We found that LEV alleviated the behavioral deficits by reducing the burden of SPs and the phosphorylation of tau. In addition, LEV enhanced the degradation and clearance of Aβ through activating autophagy and enhancing Aβ transport across the blood-brain barrier in APP23/MAPT mice. Interestingly, LEV suppressed neuroinflammation by deactivating inflammasomes and protected neurons from dystrophy and neuronal loss by suppressing KA-induced apoptosis.

As an antiepileptic drug, LEV was found to improve memory decline in APP23/MAPT mice. As AD is pathologically characterized by the presence of Aβ and tau protein in the brain, especially in the hippocampus (Ruan et al., 2019; Zheng et al., 2019), we therefore establish APP23/MAPT mice to investigate the roles of LEV in antagonizing the effects of KA on inducing the production and deposition of Aβ and p-tau. Noteworthy, selecting KA for treating APP23/MAPK mice is caused by the incidence of seizures in patients with AD (Amatniek et al., 2006). These KA-treated APP23/MAPT mice will be more closely mimicking and associated with the pathogenesis of AD patients. By these aged KA-activated APP23/MAPT mice, we demonstrated that chronic administration of LEV (L) before training partially reversed memory impairment in aged APP23/MAPT mice. Our results are in agreement with those of recent studies showing that LEV treatment reversed abnormalities in the expression of neuronal activity-related proteins that reflect hippocampal remodeling and correlate well with cognitive deficits in hAPPJ20 mice (Palop et al., 2007). Hippocampal spatial memory decline in aged rats was rescued by chronic infusion or a single injection of LEV and sodium valproate before training; this was reflected in the MWM test performance (Koh et al., 2010). Using high-resolution functional magnetic resonance imaging techniques, LEV treatment has also been shown to normalize the activity of hippocampal CA3/DG, which improved memory performance in patients with amnestic mild cognitive impairment (Bakker et al., 2012). In APP/PS1 mice, LEV shows its effects on alleviating behavioral deficits and reducing neuropathology of AD (Shi et al., 2013). Based on the above study, LEV has shown its effects on improving the neuropathology of APP/PS1 Tg mice *via* IP administration, which supports its ability to penetrate BBB (Shi et al., 2013). Along these lines, LEV exerts its functional roles in AD, but not seizure.

Based on our findings, we suggest that excess neural activity may be responsible for disrupting the processes required for encoding new information during the acquisition or early phase of memory consolidation (Wilson et al., 2005). Furthermore, hippocampal hyperactivation has been hypothesized as an aberrant condition leading to AD (Putcha et al., 2011). This hypothesis has been confirmed in the brains of APP Tg mice that exhibit memory deficits (Palop et al., 2007). In accordance with this, AD patients also have a higher incidence of seizures than those in the non-demented population, which may contribute to at least some of the cognitive impairments associated with this disease (Rao et al., 2009). These observations establish the relationship between epileptic seizures and AD, providing the evidential basis for the current study.

What mechanisms may underlie the ability of LEV to rescue memory impairment in APP23/MAPT mice? Excessive loading of Aβ has been suggested to cause aberrant excitatory neuronal activity, which results in profound remodeling of neuronal circuits through compensatory inhibitory mechanisms to counteract overexcitation in APP mice (Palop et al., 2007). Consistent with these changes, equivalent proportions of abnormally hyperactive and hypoactive neurons are found in the cerebral cortex of APP Tg mice (Busche et al., 2008). Based on previous studies, it is reasonable to suspect that chronic infusion of LEV rescues memory by inhibiting the production and deposition of Aβ in AD animals. As expected, our data revealed that high concentrations of LEV inhibited the production and deposition of Aβ, but did not affect memory deficits in APP23/MAPT mice (Figures 1, 2). LEV together with TPM has also been reported to reduce Aβ generation by inhibiting the activity of γ-secretase (Shi et al., 2013), which is consistent with our findings. The underlying mechanism involves the activation of AMPK/Akt/GSK3β pathways *in vitro* and *in vivo* (Shi et al., 2013), leading to the activation of mTOR signaling, autophagy, and Aβ clearance (Vingtdeux et al., 2011). Since the modulation of autophagy has been regarded as a new therapeutic target for the treatment of AD (Lai and McLaurin, 2012; Zare-Shahabadi et al., 2015), our data extended the previous studies to find that LEV treatment enhanced autophagy, leading to the degradation of Aβ (Figure 3A). Moreover, LEV promoted the clearance of Aβ in the brains of AD animals (Figure 3B). In line with our findings, the lack of Aβ clearance is a major cause of sporadic AD (Mawuenyega et al., 2010), which might be caused by the impaired transport of Aβ from the brain to the blood in AD animals (Banks et al., 2011). With regard to the mechanism, LRP1 and RAGE are the most important receptors for transporting Aβ across the blood-brain barrier (Deane et al., 2004). Taken together, our results suggested that LEV plays a role in increasing the Aβ clearance by upregulating LRP1 (Figure 3B).

Tau hyperphosphorylation—another hallmark of AD pathology—changes the function of tau from assembly promotion to assembly disruption, leading to impaired synaptic plasticity and axonal transport (Wang et al., 2013). Tau phosphorylation can also be induced by Aβ production and deposition (Hou et al., 2020). We found that LEV decreased the phosphorylation of tau in CDK5- and GSK3α/β-dependent mechanisms (Figure 4). Indeed, tau phosphorylation has been reported to be mediated by CDK5 and GSK3α/β (Liu S. L. et al., 2016; Maqbool et al., 2016). Importantly, cognitive impairment was alleviated by low, but not high concentrations of LEV (Figure 1). Based on these findings, we can infer that low LEV concentrations improve memory deficits by inhibiting tau phosphorylation.

Excessive Aβ production and tau hyperphosphorylation give rise to neuroinflammation and neuronal loss in AD pathology (Fu et al., 2017). Inflammasomes have been shown to contribute to various disorders in the central nervous system by causing neuroinflammation. Extracellular accumulation of Aβ in SPs in AD brains is a principal event in AD pathogenesis (Klementieva et al., 2017). Deposition of Aβ peptide initiates inflammasome activity in the microglia (Halle et al., 2008). Moreover, the activation of inflammasomes causes impairment in AD pathogenesis—through Aβ deposition and loss of spatial memory—by mediating a harmful chronic inflammatory response. It is important to note that NLRP3 activation in the brain is restricted to plaque-associated microglia, suggesting that microglial activation of the NLRP3 inflammasome is pivotal for AD pathogenesis. Furthermore, the correlation of AD pathogenesis with local neuroinflammation is already established. As an important component of the inflammasome, IL-1β has the ability to induce the phosphorylation of tau (Morales et al., 2010), leading to the compromised learning and memory in AD animals (Maccioni et al., 2009). By blocking IL-1β, the pathogenesis of AD was improved in the animal models (Murray and Lynch, 1998). In agreement with these reports, the current study further shows that LEV treatment deactivates inflammasomes, thus suppressing the activity of glial cells. To distinguish the roles of glial cells in clearing Aβ, microglial cells are responsible for degrading Aβ *via* autophagy (Cho et al., 2014), whereas astrocytes are responsible for the efflux of Aβ from the brain (Liu R. X. et al., 2016).

Synaptic dystrophy and neuronal loss are known to be significantly increased in AD mice (Pickering and O’Connor, 2007; Kitazawa et al., 2011). Our data demonstrate that a low concentration of LEV can protect neurons from synaptic dystrophy and neuronal loss, whereas high concentrations show neurotoxicity, leading to apoptosis in neurons. LEV administration after hypoxia also reduces neuronal apoptosis in a neonatal rat model of hypoxic-ischemic brain injury (Del Bo et al., 1995). Additionally, a study showed that LEV conferred neuroprotective effects against focal cerebral ischemia-reperfusion injury in mice (Brun and Englund, 1981). Unfortunately, the authors of that study did not measure the effects of different LEV concentrations on neuronal apoptosis. Here, we extended the previous investigations and found that LEV (H) induced apoptosis in neurons.

## Data availability statement

The original contributions presented in this study are included in the article/supplementary material, further inquiries can be directed to the corresponding author/s.

## Ethics statement

The animal study was reviewed and approved by Animal Care and Use Committee of The First hospital of Jilin University, Changchun, China.

## Author contributions

X-YZ and YR conceived the project and wrote the manuscript. H-CZ, Y-DL, F-YJ, X-JW, and JZ conducted the research or assisted the research, discussed the project, and assisted the manuscript preparation. JZ supervised the project. All authors are accountable for all aspects of the work and all persons designated as authors qualify for the authorship, all those who qualify for authorship are listed, and read and approved the final version of the manuscript submitted for publication.
